# Green Extraction Methods for Active Compounds from Food Waste—Cocoa Bean Shell

**DOI:** 10.3390/foods9020140

**Published:** 2020-01-30

**Authors:** Nika Pavlović, Stela Jokić, Martina Jakovljević, Marijana Blažić, Maja Molnar

**Affiliations:** 1 Faculty of Medicine Osijek, Josip Juraj Strossmayer University of Osijek, Josipa Huttlera 4, 31000 Osijek, Croatia; nika.felicita@gmail.com; 2 Faculty of Food Technology Osijek, Josip Juraj Strossmayer University of Osijek, Franje Kuhača 20, 31000 Osijek, Croatia; 3 Karlovac University of Applied Sciences, Trg J. J. Strossmayera 9, 47000 Karlovac, Croatia; marijana.blazic@vuka.hr

**Keywords:** cocoa bean shell, waste, deep eutectic solvents, microwave-assisted extraction, active compounds

## Abstract

This is the first report on the extraction of cocoa bean shell (CBS) using deep eutectic solvents (DESs). Screening results with 16 different choline chloride-based DESs showed how choline chloride:oxalic acid DES was the most suitable solvent for the extraction of the bioactive compounds from CBS and that concentrations varied greatly depending on the used solvent. The DES extraction was compared to the DESs coupled with microwave extraction (MAE), and the yields of the extracted compounds were higher for DES/MAE. For theobromine, the obtained yields for DES extraction were 2.145–4.682 mg/g, and for caffeine, were 0.681–1.524 mg/g, whereas for DES/MAE, the same compounds were obtained in 2.502–5.004 mg/g and 0.778–1.599 mg/g. Antioxidant activity was also determined, using DPPH method, obtaining 24.027–74.805% activity for DES extraction and 11.751–55.444% for DES/MAE. Water content significantly influenced the extraction of targeted active compounds from CBS, whereas extraction time and temperature did not show statistically significant influence. The extraction temperature only influenced antioxidant activity. The study demonstrated how extraction using DES and microwaves could be of a great importance in the future trends of green chemistry for the production of CBS extracts rich in bioactive compounds.

## 1. Introduction

To minimize food waste during processing of raw materials, a new age tendency is to transform it into valuable products with certain nutritional value. Cocoa bean shell (CBS) is just one of these food industry by-products, which contains a number of bioactive compounds that can be extracted and used for different purposes. In the production of chocolate and its products, the CBS is removed from the cocoa bean cotyledons, during the pre-roasting or after the roasting process. In the fermentation, roasting, and alkalization process, some bioactive compounds, like theobromine and phenols, can migrate from cocoa bean to CBS [1,2]. Thus, enriched CBS could potentially be an interesting ingredient in other production processes [1].

The chemical composition of CBS mostly depends on the origin of *Theobroma cacao* L. and its processing. Okyama et al. [2] in their review study mentioned Africa, America, Asia, and Oceania as the major parts in the world for the production of cocoa beans. 

The basic composition, however, includes dietary fibers; carbohydrates; methylxantines, like theobromine (3,7-dimethylxantine), caffeine (1,3,7-trimethylxantine), and theophylline (1,3-dimethylxantine); fats; and some phenolic compounds. CBS possesses a high nutritive value, but due to the high theobromine and caffeine content, its utilization in animal feeds is limited [3,4,5]. Nevertheless, it has been used as a goat feed [6], fish feed [7,8], pig feed [9], and as adsorbent for wastewater treatment [10]. Some authors used it as a fortification agent in corn snack products [11], whereas it has also been used as a flour in muffin and biscuit production [12,13]. CBS extracts can also find a numerous interesting applications, such as the application in oral care, due to their anti-cariogenic activity [14] and inhibition of plaque deposition [15]. As the CBS is rich in fibers and has a certain nutritional value, it was demonstrated that its nutritional effects include a good effect of children idiopathic constipation [16] and reduce the food intake as well as body weight gain [12,13]. Therefore, its potential applications in food industry are numerous. Theobromine, as one of the most abundant compounds of CBS, possesses some pharmacological properties such as diuretic, cardiac stimulant, smooth-muscle relaxant, anticancer, hypocholesterolemic, asthma, and coronary vasodilator activity [3], whereas caffeine and theophylline possess a similar psychoactive activity [2]. Caffeine accelerates the heart rate and raises blood pressure, whereas adverse effects could be manifested as insomnia, nervousness, tachycardia, arrhythmia, elevated respiration, and gastrointestinal disturbances [17]. The main fatty acids in CBS are palmitic, stearic, and oleic acid, similar to the fat content of cocoa butter, and also with twice as much of linoleic acid in CBS [2]. Most abundant polyphenols in CBS are epicatechin and catechin [18] and some phenolic acids like gallic and chlorogenic acid [19].

All of the above-mentioned active compounds can be extracted from the CBS employing various extraction tehniques. Okiyama et al. [20] extracted catehin, epicatehin, theobromine, and caffeine by pressurized liquid extraction with ethanol. Karim et al. [21] performed an extraction of CBS by aqueous ethanol to determine an antioxidant potential of such extracts, whereas Grillo et al. [22] extracted theobromine and caffeine using 30:49:21 Hex/EtOH/H_2_O mixture. Nguyen and Nguyen [23] were able to extract theobromine from the cocoa shell with 70% ethanol, and to obtain the highest possible yield, the purification was carried out with 10% lead acetate solution. Arlorio et al. [24] found caffeine in CO_2_ extracts of CBS. Jokić et al. [19] explored subcritical water extraction (SWE) of CBS to extract specific bioactive compounds. Pavlović et al. [25] provided insight into the bioactive compound profile in CBS extracts, obtained by supercritical CO_2_ extraction, ultrasound-assisted extraction (UAE), high-voltage electric discharge extraction (HVED), and DESs. This research is a continuation of our efforts in the application of green technologies in extraction of plant bioactive compounds, more specifically in extraction of bioactive compounds from CBS.

To achieve the most efficient extraction for the selected compounds, critical input parameters should be included: the nature of plant matrix, chemistry of bioactive compounds of our interest, used solvent, applied process parameters, as well as scientific expertise [26]. The environmentally friendly character of deep eutectic solvents (DESs) is accomplished through the utilization of nontoxic, biodegradable, cheap compounds that are used in their formation, as well as the DESs low vapor pressure and recyclability. It is a mixture of two or more compounds with a lower melting point than the melting points of each compound individually. One of the interesting features of these types of solvents is the possibility of making a large number of eutectic mixtures with different chemical and physical properties, due to changing one or both compounds [27]. In general, DESs have shown a great potential as the extracting solvents for different bioactive compounds from various plants. DESs have already been successfully utilized in the extraction of phenolic compounds from olive oil [28], from grape skin [29], phenolic compounds from *Barsonima intermedia* leaves [30], rutin from *Sophora japonica* [31], or *Ruta graveolens* [32]. Due to the scarcely applied DES extraction of some alkaloids from herbal medicine, Jiang et al. [33] tailored 75 types of DESs for the extraction of bioactive alkaloids and remarked how the developed DES extraction method was efficient and green. DES can be successfully utilized as such or in a combination with microwave-assisted extraction (MAE) [27]. Azmir et al. [26] mentioned studies describing some MAE advantages like quicker heating, reduced thermal gradients and increased extract yield. In DES/MAE extraction, DES acts in the way to absorb microwave radiation and to disrupt cell walls, which enables compounds to release from the sample matrix. This uniform energy transfer gives a shorter extraction time as well as low solvent consumption, which makes it a powerful separation technique [34].

To address the environmentally friendly extraction protocols, we have employed DESs for the extraction of different constituents of CBS, as well as the combination of DESs and MAE extraction techniques on the same matrix. In distinction from the existing papers, the present research provides novelty with respect to (a) detail extraction of cocoa bean shell (CBS) using deep eutectic solvents (DESs) enhanced by microwave-assisted extraction (MAE); (b) High Performance Liquid Chromatography (HPLC) analysis of methylxantines and polyphenols in obtained extracts; and (c) optimization of the main extraction parameters using response surface methodology (RSM), based on the antioxidant capacity and the concentration of each individually selected active compound.

## 2. Materials and Methods

### 2.1. Material

Cocoa bean shell (CBS) material was obtained from Kandit d.o.o. Chocolate Factory, Osijek, Croatia, in the summer of 2017. Before it was delivered from the factory, CBS was primarily separated from the cotyledon after the roasting process which was done at 135 °C for 55 min. The countries of geographical origin of CBS were Ghana, Cameroon, Nigeria, New Guinea and Ivory Coast and this is known as West Africa blend.

All chemicals (standards and organic solvents) were purchased from commercial suppliers and were of analytical grade. Solvents were purchased from J.T. Baker (Phillipsburg, USA). All standards for HPLC analysis, including theobromine standard (purity ≥98%), theophylline (purity ≥99%), gallic acid (purity ≥99%), epicatechin (purity ≥98%), catechin (purity ≥99%), and caffeic acid (purity ≥99%), were purchased from Sigma Aldrich (Germany), whereas the caffeine standard (≥98%) was purchased from Dr. Ehrenstorfer (Augsburg, Germany). DPPH radical was purchased from Sigma-Aldrich (St. Louis, MI, USA).

### 2.2. Preparation of Deep Eutectic Solvents (DES)

DESs were prepared according to our previous work [35]. Choline chloride as a hydrogen bond acceptor (HBA) was mixed with different hydrogen bond donors (HBD), as indicated in the Table 1. The mixture was stirred and heated at 80 °C until a clear liquid was formed and used in further extraction experiments as such.

### 2.3. Extraction of Bioactive Compounds from CBS

Prior to the extraction, CBS was grounded on the standard laboratory mill. The extraction of CBS was performed by weighting 50 mg of the grinded CBS in the centrifuge tube vial and adding 1 mL of the solvent (10% or 50% H_2_O/DES mixture). The extraction was performed at 50 °C during 60 min and by stirring. Afterwards, the extracts were centrifuged and decanted. Screening analysis was performed in sixteen different choline chloride-based DESs, where choline chloride was combined with different hydrogen bond donors according to the Table 1. Furthermore, when the best DES was chosen for this type of extraction, 17 runs, including five replicates, were performed according to the Box-Behnken design by RSM (Table 2) [35].

### 2.4. DESs Extraction Coupled with MAE

The extraction of bioactive compounds from *Theobroma cacao* L. CBS, a byproduct in the production of cocoa and its products, by DES extraction coupled with microwave-assisted extraction (MAE) was performed using Milestone flexiWAVE (Milestone Srl, Sorisole (BG), Italy) microwave system, equipped with rotating carousel with 15 positions for PTFE high-pressure vessels. The microwave power was set to 600–800 W. A 500 mg of CBS was weighted in the high-pressure vessel, followed by the addition of 10 mL of the solvent (10% or 50% H_2_O/DES mixture). All extraction experiments were performed at the same extraction conditions as the DES extraction with stirring (Table 3).

### 2.5. HPLC Analysis

Identification and quantification of bioactive compounds in CBS extracts was done according to modified Alves de Oliveira Nascimento et al. [36] study. After the extraction, and prior to HPLC analysis, extracts were diluted 1:10 with water and filtered through 0.2 µm polytetrafluoroethylene (PTFE) syringe filter. The measurement was done on the High-Performance Liquid Chromatograph (HPLC) (Infinity 1260 Agilent Technologies, Santa Clara, USA), which contained an autosampler G7129A, quaternary pump G7111B 1260, and diode array detector (DAD) G7117C 1260 DAD HS. A column used was Zorbax C_18_ 150 mm x 4.6 mm x 5µm, which was thermostated on 30 °C. The wavelength used for the measurement was 276 nm while the injection volume was set to 20 µL. A gradient mobile phase was used, starting by 1% formic acid and acetonitrile (95:5) at the beginning, changing to (80:20) till 9 min, and returning to (95:5) till 13 min. The flow of the mobile phase was set to 1 mL/min, and the analysis was performed in triplicate.

### 2.6. DPPH Scavenging Activity

Antioxidant activity of the obtained CBS extracts was determined using DPPH radical. The solution mix for the measurement was prepared by adding 1.2 mL of a CBS extract (10 mg/mL) and 0.5 mL of 0.2 mM DPPH solution freshly prepared in methanol.

After 30 min of incubation in the dark and at ambient temperature, the apsorbance (A) was measured at 517 nm on the UV–Visible Spectrophotometer (Termo Spectronic, Cambridge, Great Britain) and DPPH scavenging activity was determined according to the Equation (1):

(1) $$\%\;DPPH=\frac{\left(A_{DPPH}+A_{b}\right)-A_{s}}{A_{DPPH}}*100$$

where *A*_DPPH_ was the absorbance of the control (instead of the sample, methanol was added); *A*_b_ was the absorbance of the sample, where instead of DPPH methanol was added and *A*_s_ was the absorbance of the sample mixed with DPPH radical solution [37]. All measurements were performed in triplicate.

### 2.7. Statistical Analysis

The commercial Design-Expert^®^ software (ver. 9, Stat-Ease Inc., Minneapolis, MN, USA) was used for the statistical analysis of obtained experimental data. To estimate the quality of the obtained models, the analysis of variance (ANOVA) was used. The test of the statistically significant difference was based on the total error criteria with the level of confidence of 95.0%. The response plots were generated using the same software for better understanding the correlation of independent and response variables.

## 3. Results

### 3.1. DES Extraction of Bioactive Compounds from CBS

This research is the first detailed report on the extraction of CBS using DESs. In our previously study [25], only a short insight into DES extraction was given, where only one DES was described, namely, choline chloride:oxalic acid (0, 25, and 50% of water and 60, 180, and 360 min). There we indicated that the time of 60 min is sufficient for the extraction of bioactive compounds from plant material (theobromine 3.700 mg/g and 3.644 mg/g, and caffeine 0.507 mg/g and 0.536 mg/g with 25% and 50% H_2_O), whereas the energy consumption during heating is also reduced in shorter time. Furthermore, some of the DES possess a high viscosity at room temperature, which can be reduced by the increased temperature and the addition of water. Both thermolabile compounds and DES can be degraded at high temperatures, so the temperature of 50 °C was applied. Hereby, in this paper, we investigated a larger number of DESs, followed by the optimization of the process parameters.

In general, DESs can be formed from various HBAs and HBDs, and their combination, as well as the ratio, affects their physical and chemical properties greatly. Due to the different chemical and physical properties, they can exhibit a different extraction potential of various bioactive compounds. Therefore, an extraction of selected compounds in this study was performed in 16 different DESs. Our research was conducted utilizing choline chloride based DESs, where choline chloride was combined with different HBDs, as indicated in the Table 1. A screening was performed with the addition of water to each DESs (10% and 50% *v*/*v*) for 60 min, at temperature of 50 °C.

A screening experiment revealed that the extracted amount of bioactive compounds varied greatly depending on the type of the solvent, as expected. Gallic acid was extracted only with ChCl:AA (0.009 and 0.016 mg/g), ChCl:EG (0.008 and 0.002 mg/g) and ChCl:Glu (0.009 mg/g). The highest content of theobromine was extracted with ChCl:BD (3.639 mg/g), ChCl:OA (3.605 mg/g), and ChCl:U (3.613 mg/g) with 50% of water, whereas the lowest amount of theobromine was extracted with ChCl:OA (0.620 mg/g) with 10% of water. Catechin was extracted in the highest amount with ChCl:Fru (0.095 mg/g) with 50% of added water, whereas ChCl:MA, ChCl:Lev, ChCl:CA, ChCl:Mal, ChCl:LA, ChCl:OA, and ChCl:TA yielded no catechin. Caffeine was found in the highest amount when extracted with ChCl:OA (0.909 mg/g) with 50 % of water and ChCl:Lev (0.916 mg/g) with 10 % of water, and in the lowest amount in ChCl:OA (0.156 mg/g) with 10 % of water. Caffeic acid was the most abundant in the ChCl:Sor (0.04 mg/g) with 50% of water and in the lowest concentration with ChCl:OA (0 mg/g) with 10% of water. The amount of epicatechin was the highest in ChCl:Mal (0.114 mg/g) with 50% of water, whereas the lowest was in ChCl:OA (0 mg/kg) with 10% of water. Futhermore, the best antioxidant activity, expressed as DPPH scavenging activity, was obtained with ChCl:OA, 66.307% with 50% of water and 64.322% with 10% of water. When compared to the ultrasound assisted water extraction of CBS, performed in our previous research [25], obtaining theobromine yield of 4.44–5.61 mg/g and caffeine content of 0.537–0.591 mg/g, we can conclude that DESs possess a comparable efficacy for the extraction of selected compounds.

Given all the data, and the fact that some compounds are not always desirable in the obtained extracts, we have decided to optimize the extraction conditions of selected compounds in ChCl:OA DES. This DES was chosen because the influence of the water addition on the amount of bioactive compounds was the highest, thus being the most suitable for tuning the parameters for extraction of desired compounds. Therefore, both CBS extracts, with either high or low theobromine or caffeine content, can be obtained with the same solvent, only varying the water content and based on this parameter one can tune the desirable properties of the selected DES. Furthermore, the capability of the solvent to extract different undesirable compounds from the material, offers an opportunity for these solvents to be used in the pretreatment process of the material prior to its further utilization for different purposes.

The optimization of the extraction parameters was performed in a combination of 17 different experiments according to BBD, varying the temperature, extraction time, and the amount of water. Furthermore, for comparison, all experiments were performed by stirring (Table 2) and under microwaves (Table 3), applying the same DES and process parameters.

Above all, the results represented in Table 2 and Table 3 showed how DES extraction coupled with MAE gave slightly higher concentrations of selected bioactive compounds in CBS extracts. Some compounds that were found in traces after DES extraction, gave better yields after using DES/MAE, like gallic acid. These results were expected, as it is well established that the application of microwaves in many chemical processes, including extraction, can enhance the yield, shorten the reaction time, and reduce the overall energy consumption. This is well explained by the thermal and non-thermal effects of the microwaves explained in detail by de la Hoz, Diaz-Ortiz, and Moreno [38]. Furthermore, by observing the obtained concentrations of those bioactive compounds (Table 2 and Table 3) it is noticeable how different parameters variously influence on the amount of every specific compound. The highest theobromine and caffeine concentrations were found by applying 60 °C and 30% of water during 10 min of extraction time (5.004 mg/g and 1.599 mg/g), as well as the highest DPPH scavenging activity (55.444%). There are many data in the literature describing the amount of available theobromine in the CBS. Adamafio [39] stated in his review how theobromine content in CBS can vary from 5–21 mg/g according to different authors. Arlorio et al. [40] determined the amount of theobromine in cocoa hulls, which was 12.9 ± 1.8 mg/g_d.w._, whereas Sotelo and Alvarez [41] performed a water extraction and determined that the amount of theobromine in CBS was 0.223 mg/g of dry sample in fermented samples and 0.174 mg/g in not fermented samples, while caffeine was 0.056 mg/g in fermented, and 0.051 mg/g in non-fermented samples. Prabhakaran Nair [42] claims that the amount of theobromine in CBS is 1.3 % and caffeine 0.1 %. Okiyama et al. [20] performed a pressurized liquid extraction with absolute ethanol and found that the amount of theobromine was 9.89 ± 0.09 mg/g, epicatechin 3.5 ± 0.1 mg/g, caffeine 1.44 ± 0.01 mg/g, and catechin 0.178 ± 0.003 mg/g. The highest content of caffeic acid in our research, was found by applying 30 °C and 50% of water content, during 10 min of extraction time (0.137 mg/g), whereas catechin and epicatechin were 0.065 and 0.107 mg/g, respectively. 

### 3.2. Response Surface Analysis and Process Optimization

To investigate the influence of the process parameters on the extraction yield and antioxidant activity of selected compounds a response surface analysis was performed.

As gallic acid, theobromine, catechin, caffeine, and caffeic acid were obtained in higher yields applying DES with microwaves, the ANOVA was performed considering the DES/MAE of methylxantines, catechins, and antioxidant activity. The results are summarized in Table 4, Table 5, Table 6 and Table 7. For theobromine, only effect of water content in DES was significant (*p* ˂ 0.05) in first order linear and quadratic effect (*X*_3_ and *X*_3_^2^). The model F-value of 5.77 implies the model is significant, and a *p*-value of 0.0153 indicates that model terms are significant (Table 4).

Catechin shows the same trend as theobromine, where only the linear effect of the water content (*p* = 0.0302) as well as the quadratic effect of the temperature (*p* = 0.0059) had a significant influence (Table 5). The model F-value of 3.80 implies the model is significant. There is only a 4.61% chance that an F-value this large could occur due to noise. *p*-values less than 0.05 indicate model terms are significant like in this case. The Lack of Fit F-value of 2.20 implies the Lack of Fit is not significant relative to the pure error. Nonsignificant lack of fit (*p* = 0.2306) is good and implies the good model fitting.

The model F-value of 10.01 implies the model for caffeine is significant (Table 6). There is only a 0.31% chance that an F-value this large could occur due to noise. The model *p*-value is less than 0.05, indicating that model is statistically significant. In this case, the linear term of water content (*X*_3_) as well as quadratic term (*X*_3_^2^) are significant model terms for caffeine content. The Lack of Fit F-value of 2.41 implies the Lack of Fit is not significant relative to the pure error. There is a 20.71% chance that a Lack of Fit F-value this large could occur due to noise. Nonsignificant lack of fit in the case of caffeine is desirable.

ANOVA for antioxidant activity (Table 7) in CBS extracts show that model is significant (*p* = 0.0211) and that linear term of temperature (*X*_1_) as well as quadratic term of temperature (*X*_1_^2^) and time (*X*_2_^2^) influenced significantly of investigated response. In this case, water content had no significant influence. The lack of fit F-value of 1.52 implies the lack of fit is not significant relative to the pure error.

Summarizing all the obtained results, it can be concluded that water content had the strongest influence on all investigated active compounds in CBS extracts (*p*-value is ˂0.05), whereas the extraction temperature only influenced an antioxidant activity.

**Figure 1:**
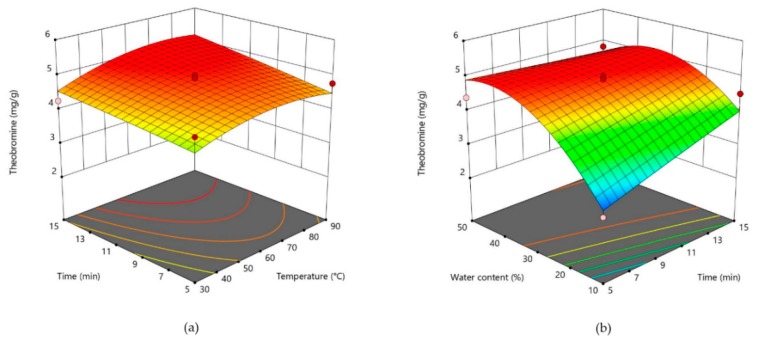
Three-dimensional plot for theobromine content in cocoa shell extracts as a function of extraction time and temperature (**a**). Three-dimensional plot for theobromine content in cocoa shell extracts as a function of water content and extraction time (**b**).

**Figure 2:**
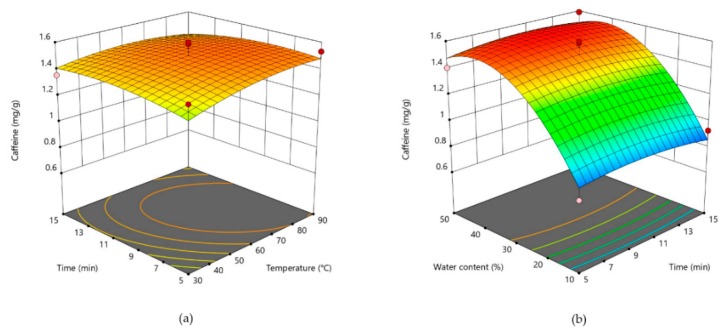
Three-dimensional plot of caffeine content in cocoa shell extracts as a function of extraction time and temperature (**a**). Three-dimensional plot of caffeine in cocoa shell extracts as a function of water content and extraction time (**b**).

**Figure 3:**
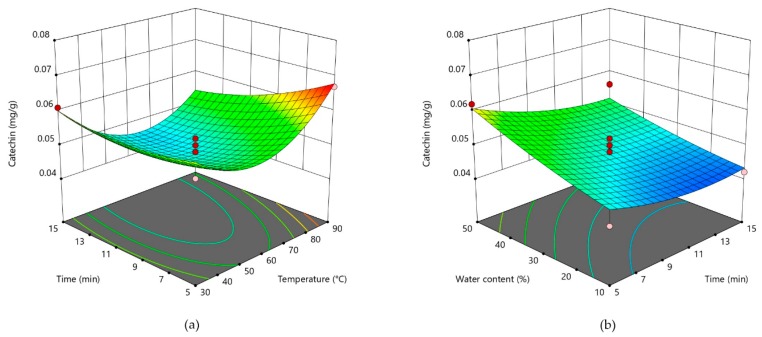
Three-dimensional plot of catechin content in cocoa shell extracts as a function of extraction time and temperature (**a**). Three-dimensional plot of catechin in cocoa shell extracts as a function of water content and extraction time (**b**).

**Figure 4:**
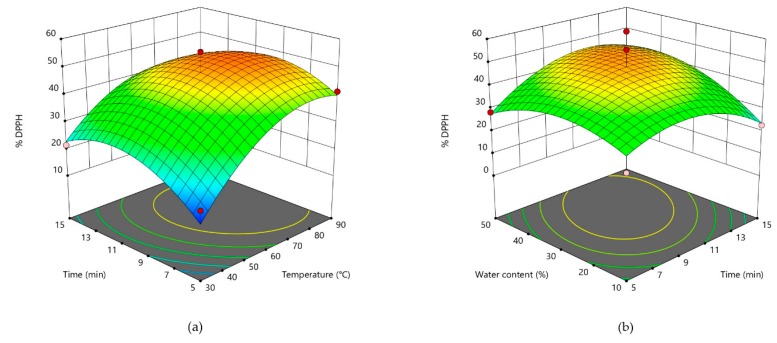
Three-dimensional plot of % DPPH in cocoa shell extracts as a function of extraction time and temperature (**a**). Three-dimensional plot of % DPPH in cocoa shell extracts as a function of water content and extraction time (**b**).

Three-dimensional plots are given for theobromine in Figure 1, for caffeine in Figure 2, and for catechin in Figure 3. For two most abundant methylxanthines in CBS three dimensional plots (Figure 1 and Table 2) showed very similar shapes. The plots show that by increasing water content, concentration of those two compounds significantly increase. As theobromine has a limited solubility in water [23], two opposite effects of water probably influenced its extractability. It could be assumed that the higher content of water decreases theobromine extractability due to its lower solubility, whereas at the same time, it causes a lower viscosity of the solvent, thus increasing its diffusion from the CBS. In addition, when water is added in higher amounts to the DES, it decreases a hydrogen bond formation between DES and the bioactive compound, thus decreasing its extractability [43]. Opposite to that, temperature and extraction time had no significant influence on methylxantine concentrations in obtained CBS extracts. All three parameters (water content, time, and temperature) significantly influenced on the DPPH scavenging activity (Figure 4). The increased DPPH activity can be attributed to the higher amount of antioxidants in the extracts. Bajkacz et al. [44] stated that the increase in water content (more than 10%) in DES increased the flavonoid recovery from food samples. According to them, the best yields were obtained with 30% water content, while the further increase up to 75% decreased the flavonoid content.

The equations, in terms of coded factors (Table 8), can be used to make predictions about the response for given levels of each factor. By default, the high levels of the factors are coded as +1 and the low levels are coded as −1. The coded equation is useful for identifying the relative impact of the factors by comparing the factor coefficients.

The optimization procedure is the fundamental tool in extraction processes; therefore, in this study, considering the maximum and by applying desirability function method, the optimal conditions for DES/MAE of CBS were estimated to be at time 11.410 min, temperature 35.106 °C, and using water content 49.392 %. Under these optimal conditions given by desirability function method and considering the maximum, theobromine concentration would be 4.485 mg/g, caffeine 1.509 mg/g, catechin 0.0654 mg/g, caffeic acid 0.137 mg/g, and DPPH scavenging activity 36.042%. Predicted data were experimentally verified with good agreement between predicted and experimental values with a deviation of ± 5%, which confirms significance of proposed model.

## 4. Conclusions

The combination of extraction with DESs and MAE of CBS for the purpose of isolation targeted bioactive compounds proved to be highly efficient. The results showed how only the water content in DES significantly influenced the extraction of methylxantines (theobromine and caffeine) from CBS, whereas the linear effect of the water content as well as the quadratic effect of the temperature had a significant influence on catechin. On the DPPH scavenging activity, linear and quadratic terms of temperature as well as quadratic term of time have statistically significantly influence. The study proves how these novel green extraction methods could definitely be included in the future trends of supplying and producing enriched extracts especially from food by-products like CBS which accumulation is increasingly problem in today’s world. Obtained extracts rich in various bioactive compounds could further be used as a raw material in food, pharmaceutical, and chemical industries.

## Figures and Tables

**Table 1:** Screening results for different Deep Eutectic Solvents (DESs) at different % of water, at constant time of 60 min and temperature 50 °C.

HBD	DES	Mole Ratio HBA:HBD	% H_2_O	Galic Acid (mg/g)	Theobro-Mine (mg/g)	Catechin (mg/g)	Caffeine (mg/g)	Caffeic Acid (mg/g)	Epicatechin (mg/g)	% DPPH
AA	ChCl:AA	1:2	10	0.009	3.020	0.052	0.775	0.024	0.031	33.582
			50	0.016	3.003	0.062	0.776	0.029	0.029	44.741
BD	ChCl:BD	1:2	10	0.000	2.282	0.000	0.571	0.023	0.051	34.280
			50	0.000	3.639	0.063	0.868	0.034	0.028	53.401
EG	ChCl:EG	1:1	10	0.008	3.019	0.057	0.766	0.025	0.019	50.521
			50	0.002	3.133	0.055	0.715	0.028	0.022	52.944
Fru	ChCl:Fru	1:1	10	0.000	2.062	0.051	0.560	0.026	0.039	48.911
			50	0.000	3.414	0.095	0.752	0.037	0.031	53.067
Gly	ChCl:Gly	1:2	10	0.000	2.786	0.064	0.734	0.031	0.027	30.414
			50	0.000	3.380	0.062	0.763	0.036	0.033	55.004
Glu	ChCl:Glu	1:1	10	0.00	2.086	0.048	0.546	0.024	0.034	27.308
			50	0.009	2.413	0.000	0.657	0.031	0.039	52.499
MA	ChCl:MA	1:1	10	0.000	1.017	0.00	0.207	0.011	0.000	41.997
			50	0.000	2.655	0.00	0.639	0.030	0.066	50.318
Xy	ChCl:Xy	1:1	10	0.000	1.362	0.000	0.326	0.016	0.033	23.632
			50	0.000	3.052	0.074	0.684	0.029	0.029	24.076
Lev	ChCl:Lev	1:1	10	0.000	3.382	0.000	0.916	0.034	0.057	43.438
CA	ChCl:CA	1:1	50	0.000	3.032	0.000	0.706	0.027	0.071	45.176
Mal	ChCl:Mal	1:1	10	0.000	2.773	0.000	0.701	0.023	0.055	42.647
			50	0.000	3.600	0.000	0.865	0.031	0.114	48.040
LA	ChCl:LA	1:1	10	0.000	3.145	0.000	0.790	0.036	0.059	39.654
			50	0.000	2.854	0.000	0.665	0.026	0.063	51.524
OA	ChCl:OA	1:1	10	0.000	0.620	0.000	0.156	0.000	0.000	64.322
			50	0.000	3.605	0.000	0.909	0.019	0.104	66.307
Sor	ChCl:Sor	1:1	50	0.000	3.008	0.062	0.635	0.040	0.110	32.153
U	ChCl:U	1:2	10	0.000	2.329	0.000	0.665	0.028	0.022	31.148
			50	0.000	3.613	0.069	0.848	0.037	0.011	50.462
TA	ChCl:TA	1:1	10	0.000	2.056	0.000	0.642	0.026	0.030	46.298

DES: Deep eutectic solvent; ChCl: Choline chloride; AA: Acetamide, BD: Butan 1,4-diole; EG: Ethylene glycol; Fru: Fructose; Gly: Glycerole; Glu: Glucose; MA: Malic acid; Xy: Xylitole; Lev: Levulinic acid; CA: Citric acid; Mal: Malonic acid; LA: lactic acid; OA: Oxalic acid; Sor: Sorbitol; U: Urea; TA: Tartaric acid; HBA: Hydrogen Bond Acceptor; HBD: Hydrogen Bond Donor; % DPPH: % radical-scavenging activity.

**Table 2:** Determined active compounds and antioxidant activity in extracts performed with Deep Eutectic Solvent (DES) and stirring.

RUN	T (°C)	t (min)	% H_2_O	Galic Acid (mg/g)	Theobromine (mg/g)	Catechin (mg/g)	Caffeine (mg/g)	Caffeic Acid (mg/g)	Epicatechin (mg/g)	% DPPH
1	90	15	30	0.000	4.682	0.042	1.330	0.000	0.064	74.81
2	60	10	30	traces	4.185	0.056	1.436	0.000	0.077	61.09
3	60	10	30	traces	4.241	0.054	1.400	0.000	0.069	51.26
4	90	10	10	traces	3.102	0.045	1.389	0.022	0.034	55.35
5	60	15	50	traces	4.537	0.046	1.524	0.040	0.033	42.02
6	90	5	30	traces	4.564	0.051	1.397	0.058	0.027	48.25
7	60	5	10	traces	2.145	0.035	0.681	0.056	0.030	46.79
8	60	10	30	traces	3.943	0.053	1.243	0.047	0.067	37.55
9	60	10	30	traces	4.010	0.056	1.208	0.040	0.066	33.46
10	60	10	30	traces	4.119	0.053	1.182	0.031	0.061	40.18
11	60	5	50	traces	4.024	0.051	1.252	0.064	0.017	24.03
12	60	15	10	traces	4.010	0.032	0.871	0.098	0.015	31.13
13	30	10	10	traces	2.324	0.031	0.710	0.045	0.011	37.16
14	90	10	50	traces	4.285	0.034	1.289	0.104	0.005	31.42
15	30	15	30	traces	2.630	0.051	1.185	0.073	0.000	24.22
16	30	5	30	0.000	3.195	0.054	1.084	0.043	0.000	30.35
17	30	10	50	0.000	2.877	0.053	1.032	0.047	0.000	35.80

T: temperature; t: time; % DPPH: % radical-scavenging activity.

**Table 3:** Determined active compounds and antioxidant activity in extracts obtained DES under microwaves.

RUN	T (°C)	t (min)	% H_2_O	Galic Acid (mg/g)	Theobromine (mg/g)	Catechin (mg/g)	Caffeine (mg/g)	Caffeic Acid (mg/g)	Epicatechin (mg/g)	% DPPH
1	90	15	30	0.463	4.754	0.050	1.311	0.120	0.059	32.315
2	60	10	30	0.103	5.004	0.047	1.599	0.103	0.000	55.444
3	60	10	30	0.101	4.912	0.040	1.412	0.083	0.000	55.315
4	90	10	10	0.000	3.348	0.066	0.969	0.098	0.000	48.238
5	60	15	50	0.221	4.823	0.056	1.590	0.128	0.017	48.597
6	90	5	30	0.304	4.765	0.067	1.531	0.067	0.137	41.290
7	60	5	10	0.043	2.502	0.043	0.792	0.047	0.000	24.392
8	60	10	30	0.103	4.941	0.050	1.585	0.096	0.000	43.803
9	60	10	30	0.110	4.769	0.048	1.490	0.079	0.000	42.162
10	60	10	30	0.096	4.875	0.052	1.417	0.085	0.000	43.956
11	60	5	50	0.191	4.386	0.062	1.403	0.132	0.085	28.469
12	60	15	10	0.211	4.500	0.042	0.927	0.102	0.000	22.777
13	30	10	10	0.019	2.465	0.047	0.778	0.054	0.014	11.751
14	90	10	50	0.201	4.804	0.058	1.509	0.116	0.036	35.521
15	30	15	30	0.148	4.263	0.061	1.356	0.116	0.071	21.521
16	30	5	30	0.117	4.591	0.064	1.451	0.123	0.074	17.726
17	30	10	50	0.200	4.505	0.065	1.443	0.137	0.107	24.667

T: temperature; t: time; % DPPH: % DPPH radical-scavenging activity determined according to Equation 1.

**Table 4:** Analysis of variance (ANOVA) for the response surface quadratic model for theobromine.

Source	Sum of Squares	df	Mean Square	*F*-Value	*p*-Value
Model	9.16	9	1.02	5.77	0.0153
*X*_1_-Temperature	0.4264	1	0.4264	2.42	0.1637
*X*_2_-Time	0.5492	1	0.5492	3.12	0.1208
*X*_3_-Water content	4.07	1	4.07	23.07	0.0020
*X*_1 × 2_	0.0251	1	0.0251	0.1426	0.7169
*X*_1 × 3_	0.0853	1	0.0853	0.4839	0.5091
*X*_2_ *X*_3_	0.6092	1	0.6092	3.46	0.1053
*X*_1_^2^	0.3531	1	0.3531	2.00	0.1998
*X*_2_^2^	0.0013	1	0.0013	0.0072	0.9348
*X*_3_^2^	2.90	1	2.90	16.47	0.0048
Residual	1.23	7	0.1762		
Lack of Fit	1.20	3	0.4010	52.72	0.0011
Pure Error	0.0304	4	0.0076		
Cor Total	10.39	16			
*R* ^2^	0.8813				

**Table 5:** Analysis of variance (ANOVA) for the response surface quadratic model for catechin.

Source	Sum of Squares	df	Mean Square	*F*-Value	*p*-Value
Model	0.0011	9	0.0001	3.80	0.0461
*X*_1_-Temperature	2.000 × 10^−6^	1	2.000 × 10^−6^	0.0635	0.8083
*X*_2_-Time	0.0001	1	0.0001	2.89	0.1327
*X*_3_-Water content	0.0002	1	0.0002	7.34	0.0302
*X*_1_ *X*_2_	0.0000	1	0.0000	1.56	0.2524
*X*_1_ *X*_3_	0.0002	1	0.0002	5.37	0.0537
*X*_2_ *X*_3_	6.250 × 10^−6^	1	6.250 × 10^−6^	0.1985	0.6694
*X*_1_^2^	0.0005	1	0.0005	15.24	0.0059
*X*_2_^2^	0.0000	1	0.0000	0.7862	0.4047
*X*_3_^2^	3.603 × 10^−6^	1	3.603 × 10^−6^	0.1144	0.7451
Residual	0.0002	7	0.0000		
Lack of Fit	0.0001	3	0.0000	2.20	0.2306
Pure Error	0.0001	4	0.0000		
Cor Total	0.0013	16			
*R* ^2^	0.8302				

**Table 6:** Analysis of variance (ANOVA) for the response surface quadratic model for caffeine.

Source	Sum of Squares	df	Mean Square	*F*-Value	*p*-Value
Model	1.15	9	0.1276	10.01	0.0031
*X*_1_-Temperature	0.0107	1	0.0107	0.8361	0.3909
*X*_2_-Time	6.125 × 10^−6^	1	6.125 × 10^−6^	0.0005	0.9831
*X*_3_-Water content	0.7682	1	0.7682	60.26	0.0001
*X*_1_ *X*_2_	0.0039	1	0.0039	0.3064	0.5971
*X*_1_ *X*_3_	0.0039	1	0.0039	0.3064	0.5971
*X*_2_ *X*_3_	0.0007	1	0.0007	0.0530	0.8245
*X*_1_^2^	0.0088	1	0.0088	0.6928	0.4327
*X*_2_^2^	0.0076	1	0.0076	0.5980	0.4646
*X*_3_^2^	0.3302	1	0.3302	25.90	0.0014
Residual	0.0892	7	0.0127		
Lack of Fit	0.0575	3	0.0192	2.41	0.2071
Pure Error	0.0318	4	0.0079		
Cor Total	1.24	16			
*R* ^2^	0.9279				

**Table 7:** Analysis of variance (ANOVA) for the response surface quadratic model for antioxidant activity (%DPPH).

Source	Sum of Squares	df	Mean Square	*F*-Value	*p*-Value
Model	2499.90	9	277.77	5.14	0.0211
*X*_1_-Temperature	834.36	1	834.36	15.44	0.0057
*X*_2_-Time	22.22	1	22.22	0.4111	0.5418
*X*_3_-Water content	113.21	1	113.21	2.09	0.1911
*X*_1_ *X*_2_	40.76	1	40.76	0.7541	0.4140
*X*_1_ *X*_3_	164.27	1	164.27	3.04	0.1248
*X*_2_ *X*_3_	118.20	1	118.20	2.19	0.1827
*X*_1_^2^	461.47	1	461.47	8.54	0.0223
*X*_2_^2^	376.34	1	376.34	6.96	0.0335
*X*_3_^2^	244.66	1	244.66	4.53	0.0709
Residual	378.38	7	54.05		
Lack of Fit	201.49	3	67.16	1.52	0.3389
Pure Error	176.89	4	44.22		
Cor Total	2878.28	16			
*R* ^2^	0.8685				

**Table 8:** Polynomial equations calculated after implementation of BBD (in terms of coded factors).

Regresion Coefficient	2nd Order Polynomial Equation
Theobromine (Y_1_)	4.90 + 0.2309*X*_1_ + 0.2620*X*_2_ + 0.7129*X*_3_ − 0.2896*X*_1_^2^ − 0.0173*X*_2_^2^ − 0.8301*X*_3_^2^ + 0.0793*X*_1_*X*_2_ − 0.1460*X*_1_*X*_3_ − 0.3902*X*_2_*X*_3_
Caffeine (Y_2_)	1.50 + 0.0365*X*_1_ + 0.0009*X*_2_ + 0.3099*X*_3_ − 0.0458*X*_1_^2^ − 0.0426*X*_2_^2^ − 0.2800*X*_3_^2^ − 0.0312*X*_1_*X*_2_ − 0.0313*X*_1_*X*_3_ + 0.0130*X*_2_*X*_3_
Catechin (Y_3_)	0.0474 + 0.0005*X*_1_ − 0.0034*X*_2_ *+* 0.0054*X*_3_ + 0.0107*X*_1_^2^ + 0.0024*X*_2_^2^ + 0.0009*X*_3_^2^ − 0.0035*X*_1_*X*_2_ − 0.0065*X*_1_*X*_3_ − 0.0013*X*_2_*X*_3_
DPPH (Y_4_)	48.14 + 10.21*X*_1_ + 1.67*X*_2_ + 3.76*X*_3_ − 10.47*X*_1_^2^ − 9.45*X*_2_^2^ − 7.62*X*_3_^2^ − 3.19*X*_1_*X*_2_ − 6.41*X*_1_*X*_3_ + 5.44*X*_2_*X*_3_

*X*_1_: temperature; *X*_2_: time; *X*_3_: water content
